# Innovative Behavior in the Workplace: An Empirical Study of Moderated Mediation Model of Self-Efficacy, Perceived Organizational Support, and Leader–Member Exchange

**DOI:** 10.3390/bs11120182

**Published:** 2021-12-16

**Authors:** Woo-Sung Choi, Seung-Wan Kang, Suk Bong Choi

**Affiliations:** 1 Seoul School of Integrated Sciences & Technologies, Seoul 03767, Korea; choikhan_ws@stud.assist.ac.kr; 2 College of Business, Gachon University, 1342 Seongnamdaero, Sujeong-gu, Seongnam-si 13120, Korea; 3 College of Global Business, Korea University, 2511 Sejong-ro, Sejong City 30019, Korea

**Keywords:** innovative behavior, leader–member exchange, perceived organizational support, self-efficacy, conservation of resource theory

## Abstract

Recently, most organizations, from for-profit organizations to nonprofit organizations, are facing a rapidly changing environment and increased uncertainty. Organizational performance now depends on quickly responding and overcoming change through employees’ innovative behavior. As the importance of innovative behavior has been highlighted, many organizations are looking for effective ways to encourage employees to adopt innovative behavior. From the resource perspective, innovative behavior can be regarded as high-intensity job demand, and organizations should support innovative behavior by providing and managing employees’ resources. Based on the conservation of resource perspective, this study attempted to empirically explore how self-efficacy and perceived organizational support affect the relationship between leader–member exchange (LMX) and innovative behavior. Using two-wave, time-lagged survey data from 337 employees in South Korea, we found that leader–member exchange enhances innovative behavior via the mediation of self-efficacy. Additionally, perceived organizational support positively moderates the relationship between leader–member exchange and self-efficacy. Our findings demonstrate that self-efficacy is a mediating mechanism in the relationship between leader–member exchange and innovative behavior. Furthermore, this study suggests that the higher the level of perceived organizational support, the greater the effect of leader–member exchange on innovative behavior affected by self-efficacy.

## 1. Introduction

For most organizations, innovation is no longer an option. For organizations to achieve results in an environment with never experienced changes, such as the COVID-19 pandemic, innovative behavior is desperately needed from employees who plan and implement new ideas and tasks quickly. Organizational innovation for organizational performance enhancement and survival comes from innovative behavior. Many scholars claim that innovative behavior significantly impacts organizational performance through previous studies [1,2,3,4]. This situation applies to most organizations, from for-profit organizations to nonprofit organizations. Research results have also suggested that innovation in the public service improves organizational performance, and innovative behavior increases workers’ job productivity [5,6].

Innovative behavior is the research, development, and practice of new ideas based on the mutual relationship among members in a current situation [7]. It is also defined as enhancing creativity using individual problem-solving skills in developing and implementing new ideas and strategies, products, and services [8]. Scott and Bruce [3] defined innovative behavior as an individual’s creation of an actionable plan, at the practical level, through securing the resources to discover and apply new creative ideas to achieve organizational performance. In other words, innovative behavior refers to actively creating, introducing, and applying new ideas that can help increase the performance of one’s job or group [9]. Innovative behavior is the organizational performance of an individual or group in a problem situation, such as an idea based on past experiences or an innovative idea or solution that did not exist before, and the ability to obtain support to produce a feasible model. It serves as a key factor in promoting the organization’s survival and continuously creates a competitive advantage by allowing various processes for improvement to be carried out [10]. Therefore, it may be difficult to survive in competition with an organizational culture where innovative behavior does not actively appear and does not lead to organizational innovation [11].

As the importance of innovative behavior has been highlighted, the number of studies explaining its mechanism from the conservation of resource perspective has increased. Recent empirical studies have shown that leadership style, peer support, and self-evaluation act as resources to provoke innovative behavior through employee immersion [12]. Additionally, positive psychological capital perfectly mediates the positive relationship between organizational innovation atmosphere and employees’ innovative behavior [13]. Several previous studies dealt with the relationship between employees’ innovative behavior and various leadership types, such as ethical leadership and transactional leadership [14,15]. However, not many studies have applied the conservation of resource perspective to the relationship between LMX and innovative behavior, seen as leadership based on the boss’s and subordinates’ mutual relationship, rather than a unilateral influence from the boss’s leadership style. Therefore, it is necessary to study this relationship in more detail.

From the conservation of resource perspective, innovative behavior can be viewed as a high-intensity job demand. According to Hobfoll [16], people tend to have as many resources as possible and maintain and preserve those resources. Due to this tendency, when people perceive or experience potential or actual resource loss, various stress levels rise, job dissatisfaction increases, and they experience an extreme sense of loss. Accordingly, people want to minimize their cognitive or actual resource loss and recover the perceived or actual loss through a constant effort to secure resources to maintain their own resource pool. The job demand-resource theory, which further developed discussion related to conservation of resource perspective, presents a model that can explain the phenomenon in various job performance situations. Even if there is a job demand for continuous physical and mental effort, if an employee has enough job resources to handle it, they will experience a high level of motivation. If an employee receives an excessive job demand without adequate job resources, the mental and physical burden will result in a negative experience, causing job stress [17].

Job demands include all costs required to maintain physical and mental efforts in physical, psychological, social, and organizational aspects: specifically, high job intensity, frequent emotional exhaustion in customer interactions, and role ambiguity applicable to job requirements. Job resources refer to growth, learning, and development that increase job involvement and organizational commitment. Such resources include organizational physical (wage, job security, and work environment), human or social resources (boss and co-workers, interdepartmental relationships), organizational resources (clarity of roles, participation in decision making), and job characteristics (autonomy, skill diversity, and job identity or job importance and performance feedback) [18]. In particular, Hobfoll and Lilly [19] explained that individual member’s resources play a role in motivating them to perform tasks and that self-efficacy is an individual’s job resource. In other words, employees with a high sense of self-efficacy perform more diligently and strive to acquire various job resources and job performance through this process. The higher the self-efficacy, the stronger the tendency to choose a supportive environment to achieve individual work goals, and the greater the response to external factors that support the work performance process [20].

Several studies that have employed the resource conservation theory emphasize the role and leadership of superiors as important resources to explain employees’ attitudes and behaviors. For example, empirical studies have verified the influence of LMX on employees’ job performance by employing the resource conservation theory [21] and have analyzed the relationship between LMX and employees’ innovative behavior [22]. However, it may be questioned whether it is only a superior’s responsibility to enhance employees’ innovative behavior. Based on prior studies, innovative behavior can be seen as a job performance situation including a high level of job demand, which requires various processes to solve a problem. Therefore, sufficient job resources must be acquired and retained to handle this smoothly. In addition, the quality of LMX and perceived organizational support are important factors belonging to social or organizational resources, and self-efficacy can be said to be an essential individual-level job resource based on job characteristics. Self-efficacy can also affect innovation behavior by reflecting individuals’ perceptions of social and organizational resources.

In summary, innovative behavior leads to organizational innovation and is related to organizational performance. When the organization supports innovation, employees become more interested in innovation, which becomes the basis for innovative behavior [8,23]. As Hobfoll argued, all types of job resources, such as social and organizational resources, are accumulated and used as needed in situations where job demands are high [24]. As argued by the job demand-resources theory, the motivation acquired by an individual has a positive effect on job performance [17].

LMX, a leadership variable that functions as a representative resource and perceived organizational support, which is an organizational variable, are individual variables that can amplify or weaken the influence of other resource variables while functioning as a resource by themselves. The mechanism through which self-efficacy is influenced has not been specifically studied. We were of the view that research on this topic could enhance the resource conservation theory and provide meaningful insights into the role of leadership processes and organizations in inducing innovative behavior.

Therefore, to clarify the purpose of this study again, it was intended to demonstrate the process by which LMX, perceived organizational support perception, and self-efficacy affect members’ innovative behavior in accordance with the resource conservation theory. In particular, this study is necessary to reconfirm the influence of leadership variables and to shed light on the importance of organizational variables, such as perceived organizational support and the role of organizations.

## 2. Theoretical Background and Hypotheses

### 2.1. Leader–Member Exchange (LMX) and Innovative Behavior

LMX refers to the quality of the exchange relationship formed between the leader and the members. They establish different supervisory or role relationships between the leader and the members that occur within the organization [25]. This is a concept based on the social exchange theory presented by Dansereau et al. [26].

LMX is a very complex concept related to forming in-groups and out-groups. Originally known as the vertical dyad linkage, LMX was conceptualized in the 1970s and describes the dyadic relationship between the leader and follower. LMX is rooted in the principle that leaders create differentiated relationships through the differentiated types of exchange that they have with their followers [26]. High LMX relationships are characterized by respect, trust, and a sense of mutual obligation that lead to an affective attachment for one another [21,27]. In such relationships, both the leader and follower view the relationship as socio-emotional, moving beyond a merely transactional economic exchange [28]. This leads to a reciprocal loop because when leaders show care and concern for subordinates, it creates stronger leader–follower relationships [29]. Empirically, LMX has been linked to multiple organizational outcomes [28,30].

The relationship between the leader and the members is formed over time through the process of role formation. [27,31,32]. Initially, the interaction between a leader and subordinates takes place in the performance of the formal roles defined for them; however, while these relationships continue, they eventually develop interest and effort beyond fixed roles, evolving into a noncontractual, social exchange relationship. This includes cases in which a leader requests members’ cooperation on unstructured tasks or cases in which members voluntarily take initiative to conduct activities and assume responsibilities outside of their stipulated roles. As the members accept the leader’s demands and the leader acknowledges the members’ activities outside of their roles, trust is formed as the two develop a closer relationship.

When a high-quality exchange relationship is established, both parties do their best to exchange more information, provide financial and nonfinancial support, and help each other grow in the organization. The relationship develops into a partnership characterized by mutual trust, respect, obligation, and achievement of common goals, with each person continuing to care for both work-related needs and the interests of the other [27]. In such a relationship, employees are given more job discretion, decision-making power, and an opportunity to influence operations, and they devote more effort to performing unstructured tasks. Sparrowe and Liden (2005) empirically verified that when the quality of LMX is high, a leader expands the psychological discretion of the job, such as decision-making scope, delegation of authority, feedback, and support, and establishes job autonomy [33].

Through several empirical studies, a high level of LMX has positive effects, such as promotion frequency, organizational commitment, low turnover rate, positive performance evaluation, interest and consideration from superiors, desirable work background, job attitude, and participation. It has also been suggested that the members’ productivity is higher with a higher LMX level than with a lower LMX level [34]. In addition, LMX has been confirmed to have a positive relationship with organizational citizenship behavior (OCB), which can be seen as a representative extra-role behavior [35,36].

From the perspective of the resource conservation theory, these provisions can be regarded as important job resources that can be obtained from the relationship with the leader. In other words, the support of a leader or the positive relationship between leaders and members is a representative job resource, which has a positive effect on effective job performance [37]. An empirical study applied the conservation of resource theory and suggested that LMX functions as a job resource that increases job performance by reducing employee stress [38]. Scott and Bruce (1994) revealed that LMX had a positive effect on innovation behavior [3]. Janssen and Van Yperen (2004) suggested that a high level of member perception of LMX promotes innovative behaviors that are helpful to organizational performance [39]. The more a leader delegates task-related discretion to organizational members, the greater their perception of responsibility related to task performance. Innovative behavior can be considered a high-level job demand because it involves uncertainty; therefore, it was predicted that innovative behavior would increase on the basis of job resources obtained through high interaction with leaders.

We performed the test for the main effect, which may suggest a positive relationship between LMX and innovative behavior. The positive relationship was strongly expected considering the results of previous studies on the relationship between leadership variables and innovative behavior or innovative performance [22,40]. Based on the above theoretical background and reviewing previous studies, we constructed the following hypothesis.

**Hypothesis** **1** **(H1).** *LMX is positively related to innovative behavior*.

### 2.2. Mediating Role of Self-Efficacy

Self-efficacy is the belief in an individual’s ability to organize and perform the necessary actions to execute a specific task or achieve an outcome [41]. It means a person’s beliefs, ability to motivate, cognitive resources, and the factors necessary to successfully perform a particular task in a given situation [42]. When a person with high self-efficacy faces a difficult problem, they attribute the cause to their lack of effort and continue improving their abilities. Self-efficacy forms an attitude to overcome rather than give up, even when a difficult situation arises, and it promotes a challenging response to create high job performance. Conversely, people with low self-efficacy perceive that their abilities are insufficient to achieve their goals, so they avoid or give up, even in situations where task achievement is easy [43,44].

The higher the quality of the LMX, the more formally and informally its members will gain financial, nonfinancial, and social support. In addition, high-quality LMX ensures that members receive active support, encouragement, and constructive feedback when performing their duties [27,45]. This will establish and expand the belief that members can solve even more difficult and complex problems. Mathisen (2011) also suggests that higher quality LMX is related to higher self-efficacy regarding responsibilities and expectations [46]. In addition, as Atwater and Carmeli (2009) noted, creative and innovative behavior differs from day-to-day work [47]. Innovative behavior involves considerable complexity and uncertainty; therefore, for members to perform innovative actions well, confidence in their ability to perform creative and innovative work is essential [41]. Members with a high sense of self-efficacy set more challenging goals, put more effort into achieving them, and endeavor to achieve them with patience [48]. Thus, LMX will raise self-efficacy, and self-efficacy will increase innovative behavior. In other words, it can be inferred that self-efficacy functions as a parameter in the relationship between LMX and innovative behavior.

As described above, self-efficacy can be viewed as judging whether one can successfully perform a given task [49], meaning that confidence in one’s control and utilization of factors, such as knowledge and skills necessary for task performance. Employees with high self-efficacy take more active approaches to difficult job demands. Therefore, self-efficacy is highly likely to affect job behavior by reflecting the individual’s perception of social and organizational resources. In other words, employees with a sense of self-efficacy will more actively accept job resources, such as LMX, when they are provided [50]. Additionally, individuals with high self-efficacy tend to engage in innovative behavior because they have the confidence, knowledge, and skills to generate ideas, apply them to work, and are more inclined to challenge and solve uncertainties [51]. In addition, recent empirical studies have suggested self-efficacy as an antecedent variable of innovative behavior [52,53].

A previous study on self-efficacy suggested that LMX is an important antecedent variable for self-efficacy [46]. Additionally, self-efficacy is a mediator in the relationship between LMX and creativity, which is closely related to innovative behavior [54]. Furthermore, a previous study has shown that self-efficacy has a positive relationship with innovative behavior [55]. Therefore, we can predict a positive relationship between LMX and self-efficacy and infer that self-efficacy plays a mediating role in the relationship between LMX and extra-role behaviors, such as innovative behavior. Studies have revealed that self-efficacy functions as a parameter between leadership variables such as LMX and dependent variables related to employees [56,57]. Thus, we propose the following hypothesis.

**Hypothesis** **2** **(H2).** *Self-efficacy mediates the relationship between LMX and innovative behavior, such that LMX increases employees’ self-efficacy, and the increased self-efficacy promotes employees’ innovative behavior*.

### 2.3. Moderating Role of Perceived Organizational Support

Perceived organizational support is a perception generated by organizational members concerning the level of interest in the welfare of the organization members and the values that the organization meets their expectations [58,59]. As the positive belief that members recognized by the organization are retained in the organization, high organizational support awareness strengthens emotional commitment and increases the effort provided to the organization. Conversely, when an organization is repeatedly indifferent to the contribution and welfare of its members, the members’ responsibility toward the organization is reduced [60]. 

Perceived organizational support leads to positive outcomes for employees, such as performance, commitment, and positive feelings. Employees’ belief in their competence is affected by perceived organizational support, which positively affects self-efficacy [61,62]. In addition, perceived organizational support improved organizational commitment, self-efficacy, in-role behavior, and OCB, lowering job stress, burnout, and turnover intention [63,64]. In addition, empirical studies have proven that perceived organizational support affects self-efficacy linked to job performance [65,66]. Employees try to recover actual or potential resource loss by securing resources from various sources [67]. Relationships with superiors, support from the organization, and helping coworkers function as a significant source of recovery from the loss of motivation associated with losing resources [68,69].

According to Wayne et al. (1997), LMX and POS are distinct variables. In addition, LMX and POS influence each other. This is consistent with the idea of a self-fulfilling prophecy that leaders can develop higher expectations and higher quality interactions with employees supported by the organization [70]. Employees with high levels of POS are more likely to improve their skills and abilities, which will benefit their leaders. In essence, such employees can be attractive exchange partners because they possess and pursue resources that leaders value. From the member’s point of view, resources that can be obtained from leadership variables such as LMX and resources from the organization are distinguished.

Organizational variables and leadership variables can have an interactive effect on members’ attitudes and behaviors [71]. Recognition of supervisor support is a variable that is distinct from, but closely related to, LMX [72]. Therefore, even if high-quality LMX is perceived from the perspective of subordinates, LMX and its relationship with employee attitudes, such as self-efficacy, may vary. Several studies have shown that perceived organizational support functions as a moderator variable in the relationship between leadership variables, especially LMX, and other variables [73,74,75]. 

Therefore, the interaction between LMX and perceived organizational support, which are essential job resources, is expected to affect self-efficacy. In other words, perceived organizational support can be a moderating variable in the relationship between LMX and self-efficacy. Based on these reasons, we propose the following hypothesis.

**Hypothesis** **3** **(H3).** *Perceived organizational support moderates the relationship between LMX and self-efficacy, such that the relationship will be stronger when perceived organizational support is high rather than low*.

**Figure 1:**
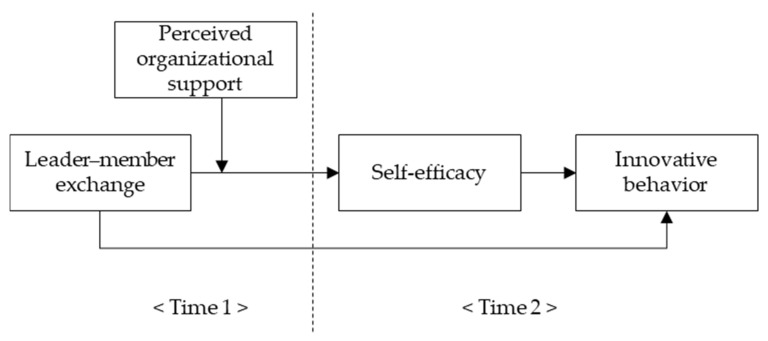
The theoretical research model.

The theoretical model of this study is depicted in Figure 1.

## 3. Methodology

### 3.1. Sample

This study minimized the possibility of a common method bias due to a cross-sectional survey [76] by dividing the variables with a time lag of 4 weeks and surveying two rounds. The 4-week time lag was chosen based on prior research, indicating that a 4-week time interval was long enough to allow for changes in employee psychological factors, such as strain, but short enough to allow for stability in one’s environment [77,78]. We recruited the target population from the Korean branch of a reliable online survey company with 45 offices in 16 countries globally specializing in academic research. The survey’s target population was randomly selected from an online panel of office workers with bosses working for South Korean companies. Before answering the questionnaires, the participants were guided through the research purpose and procedures. They were also informed of their freedom to withdraw from the survey at any time and the benefits and disadvantages that may arise from participating; they were then asked to sign an informed consent form. Data were collected only from those who signed the consent form.

The first survey was sent to 500 people via email, and a total of 420 responses were obtained, excluding unreliable responses. One month later, the second survey was sent via email to respondents who completed the first survey. Excluding unreliable responses (including incomplete responses), a total of 337 responses were collected and used for analysis (response rate: 67.4%). The sampling process is presented in Appendix A.

The demographics of the respondents were as follows: 52.8% were male, and 47.2% were female. The mean age of the respondents was 41.8 years (SD = 10.33). The respondents’ highest level of education included bachelor’s and master’s degrees (56.3%), junior college degree (28.5%), high school diploma (13.4%), and doctoral degree (1.8%). Regarding their positions, 18.1% were directors and executives in supervisory roles, and 81.9% were not. The average organizational tenure was 7.9 years (SD = 7.4).

### 3.2. Measures

The research variables’ questionnaire items were measured using a five-point Likert scale (with scores ranging from 1 = strongly not agree to 5 = strongly agree). Since the original measurement items were in English, they were translated into Korean and reviewed and corrected by experts. Then, the Korean questionnaire was translated back into English, the validity of which was verified through back translation in which the similarity of linguistic structure and meaning were compared with the original text [79]. A complete questionnaire is provided in Appendix B.

#### 3.2.1. Leader–Member Exchange (LMX)

LMX was measured using seven items developed by Scandura and Graen (1984) [32]. Meta-analytical evidence indicates that the LMX 7 provides the soundest psychometric properties and the highest correlations with outcomes in comparison to other available instruments [80]. LMX is usually a dyadic construct; however, for the purpose of the current study, we viewed LMX in relation to employees’ perceptions of the supervisor–subordinate relationship. Some examples of the questionnaire items included, “How well does your coach understand your job problems and needs?” and “I have enough confidence in my coach that I would defend and justify his or her decisions if he or she was not present to do so.” Cronbach’s alpha was 0.89.

#### 3.2.2. Perceived Organizational Support

Perceived organizational support was measured using three items as a shortened version of the SPOS developed by Eisenberger et al. (2002) [60]. Eisenberger et al. (1986) revealed Items 1, 4, and 9 of the SPOS had factor loadings of 0.71, 0.74, and 0.83, respectively, to evaluate employees’ perceptions of the organization [81]. In this study, those three items as a shortened version of the SPOS were used in accordance with results of previous studies. Some examples of the questionnaire items included, “The Organization strongly considers my goal and values,” and “The Organization really cares about my well-being.” Cronbach’s alpha was 0.85.

Some examples of the questionnaire items included, “The Organization strongly considers my goal and values,” and “The Organization really cares about my well-being.” Cronbach’s alpha was 0.85.

#### 3.2.3. Self-Efficacy

Self-efficacy was measured using three items developed by Spreitzer [82]. Some examples of the questionnaire items included, “I am self-assured about my capabilities to perform my work activities,” and “I have mastered the skills necessary for my job.” Cronbach’s alpha was 0.85.

#### 3.2.4. Innovative Behavior

Innovative behavior was measured using six items developed by Scott and Bruce [3]. Some examples of the questionnaire items included, “I develop adequate plans and schedules for the implementation of new ideas,” and “I investigate and secures funds needed to implement new ideas.” Cronbach’s alpha was 0.89.

#### 3.2.5. Control Variables

In this study, to more clearly confirm the relationship between the variables presented in the research model, gender, age, level of education, position, and organizational tenure were used as control variables. These were selected based on prior studies related to the research variables [3,83].

### 3.3. Analytical Method

We used STATA 17.0 to conduct CFA to determine the model’s validity and hierarchical regression analysis to test our research hypotheses. We followed the recommendations of Preacher and Hayes in using the bootstrapping approach to test the mediation hypothesis [84]. The moderated mediation hypothesis was analyzed by calculating the index of moderated mediation introduced by Hayes [85].

## 4. Result

The means, standard deviations, correlations, and reliability coefficients of the variables are presented in Table 1. The correlation between all the study’s key variables was statistically significant, thus providing initial evidence for all of the study’s hypothesized relationships.

### 4.1. Validity and Common Method Bias Checks

As seen in Table 2, we performed CFA to test the construct validity of study variables. The normed Chi-square (χ2 / df) was 1.61 (χ2 = 348.265, df = 216), which was less than the cut-off value of 3.00 [86]. The comparative fit index (CFI) was 0.963, and the Tucker–Lewis index (TLI) was 0.954, which exceeded the standard cut-off value of 0.95 [86]. In addition, the root mean square error (RMSEA) of approximation was 0.043, which was less than the standard cut-off value of 0.08 and even less than 0.05, which is a more desirable standard [86]. All the CFA indicators satisfied the standards verification, which we used to determine that our hypothesized measurement model was appropriate for the data. Additionally, we compared the fit of this four–factor model with three competing models, finding that the four–factor model was significantly superior to the competing models. In addition, the Average Variance Extract (AVE) and Composite Reliability (CR) values of all variables satisfied the criteria (AVE > 0.5, CR > 0.7), and the correlation values for each construct were lower than the square root of AVE [87,88]. Furthermore, all standardized factor loadings on the predicted constructs were above the cut-off value of 0.50 [89,90].

We used a two-wave, time-lagged survey to minimize the possibility of common method bias, but all variables were measured from employees’ responses. As such, Harman’s single factor test was performed in this study. The results show that the explanatory covariate of the first factor was 27.92%, and no substantial amount of common method variance was present. Therefore, the research data do not suffer from the serious issue of common method variance [76,91].

### 4.2. Hypotheses Test

To test Hypotheses 1 and 3, we implemented hierarchical multiple regression analyses. We examined and tested Hypotheses 2 and 4 by the bootstrapping analyses [84,85]. As shown in Model 5 of Table 3, LMX is significantly positively related to innovative behavior (β = 0.22, *p* < 0.001), and the explanatory power of Model 5 is significantly higher than that of Model 4 (Model 4 → Model 5: ΔR^2^ = 0.05, ΔF = 17.45, *p* < 0.001). Therefore, we determined that Hypothesis 1 is supported.

Hypothesis 2 predicted that self-efficacy mediates the relationship between LMX and innovative behavior. The bootstrapping analysis results that do not rely on the normal sampling distribution assumption show a 0.09 coefficient, and standard error is 0.02. The 95% confidence interval (CI) with 10,000 times bootstrapped samples did not include zero (low limit 0.04, upper limit 0.14). Therefore, we determined that Hypothesis 2 is supported.

**Figure 2:**
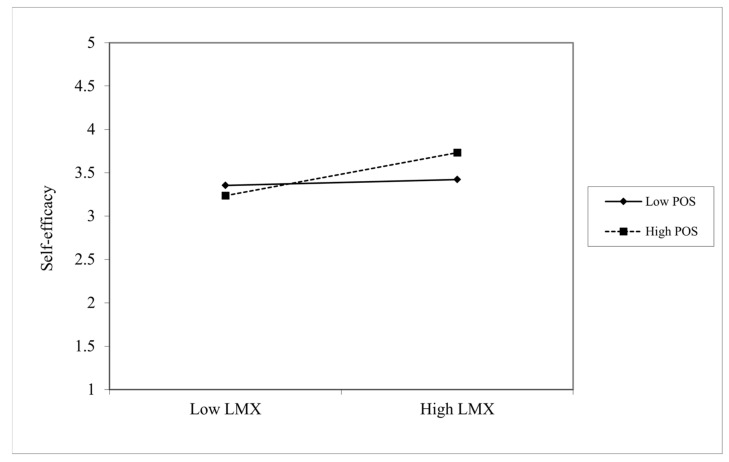
The moderating effect of perceived organizational support level on the relationship between LMX and self-efficacy. Note. LMX = leader–member exchange, POS = perceived organizational support.

Hypothesis 3 predicted that perceived organizational support moderates the relationship between LMX and self-efficacy. As shown in Model 3 of Table 3, self-efficacy is significantly positively related to the interaction of LMX and perceived organizational support (β = 0.20, *p* < 0.001), and the explanatory power of Model 3 is significantly higher than Model 2 (Model 2 → Model 3: Δ R^2^ = 0.05, ΔF = 9.35, *p* < 0.001). Furthermore, we illustrated an interpretation of the interaction pattern in Figure 2, which shows the positive relationship between LMX and self-efficacy was stronger when perceived organizational support was high. The results of the simple slope test reveal that the positive relationship between LMX and self-efficacy was significant when perceived organizational support was high (β = 0.36, *p* < 0.001) and not significant when it was low (β = 0.049, not significant) [92]. Thus, we determined that Hypothesis 3 is supported.

## 5. Discussions

### 5.1. Theoretical Contributions

This study theoretically contributes to the conservation of the resource theory and innovative behavior mechanisms by proposing several important implications. First, this study hypothesized and verified the relationship between LMX, self-efficacy, perceived organizational support, and innovative behavior, which are considered essential job resources based on the resource conservation theory. Previous studies have explained innovative behavior in the framework of the resource conservation theory; however, it was necessary to clarify the structure and relationship of related variables more clearly to materialize the theory’s application. Therefore, this study expanded the theory and contributed to accumulating in-depth knowledge regarding the relationship between variables and innovative behavior.

Second, this study revealed the mediating role of self-efficacy as a mechanism to explain the relationship between LMX and innovative behavior. This concurs with the results of previous studies that revealed that self-efficacy functions as a parameter between leadership variables such as LMX and the dependent variables related to employees [56,57]. Individuals with a strong sense of self-efficacy choose a supportive environment to achieve their work goals and respond more sensitively to external factors that help the work process. Therefore, it can be inferred that self-efficacy functions as an individual job resource and plays a role in influencing leadership, a vital job resource. One of the main interests of leadership research is to reveal the specific process of leadership influence. This study is meaningful because it inferred, based on the conservation of the resource theory, and empirically confirmed the process of LMX increasing members’ self-efficacy, leading to innovative behavior.

Lastly, this study is based on several previous studies, which have demonstrated that perceived organizational support functions as a moderating variable in the relationship between leadership variables, especially LMX, and other variables [73,74,75]. This study revealed that perceived organizational support acted as an important job resource. It was further verified that the relationship between LMX and self-efficacy was not significant when organizational support perception was low. This may have contributed to strengthening the resource conservation theory, in that resources from multiple sources such as leadership variables, organizational variables, and individual variables, are accumulated and function as resources that can respond to job demands, thus enhancing the findings of previous studies. The importance and role of the organization are highlighted, which has various implications.

### 5.2. Managerial Implications

This study’s practical implications are as follows. Firstly, by empirically demonstrating that LMX can be an antecedent factor in innovative behavior as a job resource, this study advises what kind of leadership should be suggested and taught to an organization’s leaders to encourage their members’ innovative behavior. As many scholars have argued through prior studies, innovative behavior has a significant impact on organizational performance. Leadership, especially the quality of LMX, functions as a job resource that makes employees willing to undertake innovative behaviors considered high-level job demands. Therefore, if an organization wants to innovate, it is necessary to encourage its leaders to improve the quality of their exchange relationships with subordinates and support the acquisition of capacity for this purpose.

Secondly, this study revealed the mediating role of self-efficacy as a mechanism to explain the relationship between LMX and innovative behavior. This provides insight that employees’ self-efficacy is a job resource at the individual level and that other variables, including various leadership, can play an important role in activating innovative behaviors. This provides an appropriate direction for various organizations intending to manage antecedent factors in employees’ innovative behavior.

Thirdly, this study found that when the level of organizational support awareness was low, the indirect effect of LMX on the innovation behavior of members through self-efficacy was not significant. In other words, rather than a vague expectation that the effects of LMX will be the same for members, this study found that the effects of LMX may differ depending on the situation and perception of the organization, especially the recognition of organizational support, which is the main variable of interest in this study. It provides an important implication that it is necessary to identify and effectively manage human resources.

Lastly, this study provides a lesson in the importance of human resource management, which makes employees feel supported by the organization, and relationships with leaders maximize the innovation behavior of members. This study revealed that a low level of organizational support awareness resulted in an insignificant indirect effect of LMX on the innovation behavior of members through self-efficacy. In other words, rather than a vague expectation that the LMX effect would activate for employees, corporate organizations must implement human resource management with a realistic perspective that the LMX effect can differ depending on the employees’ recognition of organizational support [93].

### 5.3. Limitations and Future Research Direction

Although this study provides meaningful implications for both scholars and practitioners, future research should consider some limitations. Firstly, although this study used research data obtained through a second survey with a time lag, it has limitations as a cross-sectional study because the measurement of each study variable was limited to each particular time point. Given the time-dependent relationship with variables, a longitudinal study that can prove a more effective causal relationship should be designed in future research.

Secondly, since this study is based on data from employees from South Korean companies only, it is possible that the cultural background significantly influenced the employees’ perceptions and attitudes. Therefore, we must be cautious when interpreting and applying our results to employees in other cultural environments.

Thirdly, because this study’s measurement of the research variables was from the same source, it is not free from concerns about the common method bias. Although staggered to separate response time points, fundamental limitations exist. As a result of the CFA, the research models’ variables were distinguished in this study, but future research should consider this issue.

Fourthly, this study considers major variables as job resources from the conservation of the resource theory perspective and demonstrates their influence on innovation behavior. In subsequent research, it is necessary to review the following research directions. Theoretically, this study examined the relationship and implications of conservation of the resource theory regarding the theoretical frameworks and perspectives, traditionally or recently applied to studies, to explain innovation behavior, such as the expectation theory, fairness theory, and social exchange theory. As such, it will be possible to provide new insights and perspectives on the application and extension of the theory.

Lastly, the organizational support perception was confirmed to act as a major moderating variable. Other organizational characteristics, such as employees’ perceptions of organizational fairness and organizational climate, may also influence other job resources on innovation behavior, such as LMX and self-efficacy. However, this study did not address this possibility. Additionally, examining the moderating function of various individual difference variables will also show meaningful results. Therefore, further research is needed to develop more detailed research models by considering different moderating variables.

## Figures and Tables

**Table 1:** Means, standard deviations, correlations, and reliabilities.

Variables	Mean	SD	1	2	3	4	5	6	7	8	9
1. Gender	0.52	0.49	-								
2. Age	41.81	10.33	0.02	-							
3. Education	2.78	1.07	0.07	−0.04	-						
4. Job level	2.58	1.53	0.36 ***	0.48 ***	0.22 ***	-					
5. Tenure	7.97	7.47	0.15 **	0.51 ***	0.09	0.43 ***	-				
6. LMX	3.26	0.68	−0.03	0.10	0.07	0.12 *	0.12 *	(0.89)			
8. POS	3.00	0.75	−0.03	0.14 **	0.04	0.12 *	0.14 **	0.43 ***	(0.85)		
9. SEF	3.67	0.60	0.01	0.08	0.10	0.11 *	0.08	0.25 ***	0.21 ***	(0.85)	
10. INB	3.31	0.64	0.03	0.15**	0.11 *	0.20 ***	0.12 *	0.25 ***	0.27 ***	0.44 ***	(0.89)

Note. N = 337, * *p* < 0.05, ** *p* < 0.01, *** *p* < 0.001, the values in parentheses denote Cronbach’s alphas, Age: year, Gender: female = 0, male = 1, Education = final level of educational: 1 = high school graduates, 2 = college graduates, 3 = university graduates, 4 = post-graduates, 5 = Ph.D. holders. Job level: 1 = staff, 2 = assistant manager, 3 = manager, 4 = senior manager, 5 = directors, 6 = executives. Tenure: organizational tenure (year), LMX = leader–member exchange, POS = perceived organizational support, SEF = self-efficacy, INB = innovative behavior.

**Table 2:** Results of confirmatory factor analysis.

Model	χ^2^(df)	CFI	TLI	RMSEA	Δχ^2^(Δdf) ^4^
Research model (4 factor)	348.265(216) ***	0.963	0.954	0.043	
Alternative model 1 (3 factor) ^1^	694.298(224) ***	0.868	0.843	0.079	346.033(8) ***
Alternative model 2 (2 factor) ^2^	1145.075(231) ***	0.743	0.704	0.109	796.81(15) ***
Alternative model 3 (1 factor) ^3^	1932.333(237) ***	0.523	0.465	0.146	1584.068(21) ***

Note. n = 377, *** *p* < 0.001, CFI = comparative fit index, TLI = Tucker–Lewis index, RMSEA = root mean square, LMX = leader–member exchange, POS = perceived organizational support, SEF = self-efficacy, INB = innovative behavior.; ^1^ 3 factor: LMX+POS, SEF, and INB, ^2^ 2 factor: LMX+POS+SEF, and INB, ^3^ 1 factor: LMX+POS+SEF+INB, ^4^ Chi-square difference for each model reflects its deviation from the four–factor model.

**Table 3:** Results of hierarchical multiple regression.

Variables	SEF			INB		
	Model 1	Model 2	Model 3	Model 4	Model 5	Model 6
Gender	−0.02	0.00	0.00	−0.03	−0.01	0.00
Age	0.03	0.03	0.00	0.08	0.08	0.06
Education	0.08	0.07	0.08	0.08	0.07	0.04
Job level	0.07	0.05	0.04	0.15 *	0.12	0.10
Tenure	0.02	0.00	0.00	0.00	0.00	−0.01
LMX		0.23 ***	0.23 ***		0.22 ***	0.07
POS			0.08			0.11 *
LMX*POS			0.20 ***			
SEF						0.38 ***
R^2^	0.02	0.07	0.12	0.05	0.10	0.25
ΔR^2^		0.05	0.05		0.05	0.15
adj R^2^	0.00	0.05	0.10	0.04	0.08	0.23
F	1.51	4.47 ***	5.86 ***	3.83 **	6.25 ***	14.12 ***
F_inc_		18.92 ***	9.35 ***	8.81 ***	17.45 ***	33.96 ***

Note. n = 337, * *p* < 0.05, ** *p* < 0.01, *** *p* < 0.001, Standardized coefficients are reported, LMX = leader–member exchange, POS = perceived organizational support, SEF = self-efficacy, INB = innovative behavior.

**Figure A1:**
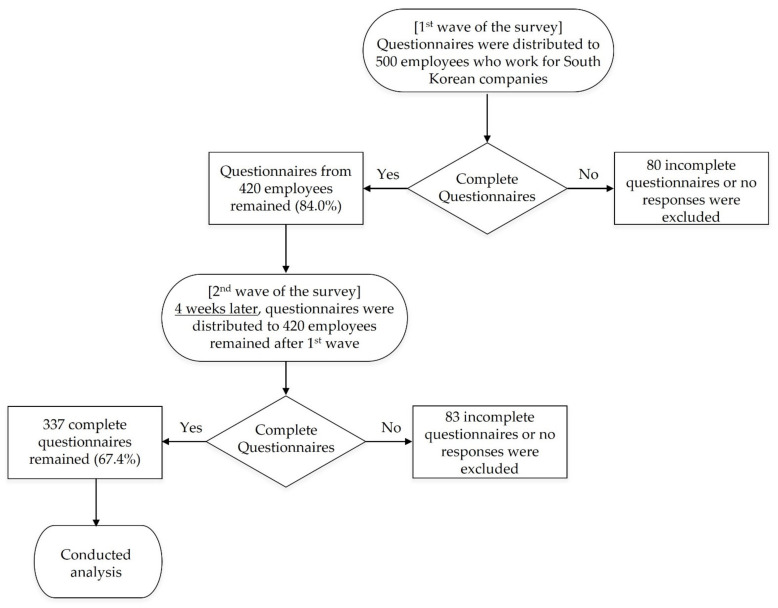
Sampling.

## Data Availability

Not applicable.

## Appendix B. Measurements

Leader–member exchange (Cronbach’s alpha = 0.89)

1. Do you usually feel that you know where you stand? Do you usually know how satisfied your immediate supervisor is with what you do?
2. How well do you feel that your immediate supervisor understands your problems and needs?
3. How well do you feel that your immediate supervisor recognizes your potential?
4. Regardless of how much formal authority your immediate supervisor has built into his or her position, what are the chances that he or she would be personally inclined to use power to help you solve problems in your work?
5. Again, regardless of the amount of formal authority your immediate supervisor has, to what extent can you count on him or her to “bail you out” at his or her expense when you really need it?
6. I have enough confidence in my immediate supervisor that I would defend and justify his or her decisions if he or she were not present to do so.
7. How would you characterize your working relationship with your immediate supervisor?

Self-efficacy (Cronbach’s alpha = 0.85)

1. I am confident about my ability to do my job.
2. I am self-assured about my capabilities to perform my work activities.
3. I have mastered the skills necessary for my job.

Perceived Organizational support (Cronbach’s alpha = 0.85).

1. The organization values my contribution to its well-being.
2. The organization strongly considers my goals and values.
3. The organization really cares about my well-being.

Innovative behavior (Cronbach’s alpha = 0.89).

1. I search out new technologies, processes, techniques, and/or product ideas.
2. I generate creative ideas.
3. I promote and champions ideas to others.
4. I investigate and secures funds needed to implement new ideas.
5. I develop adequate plans and schedules for the implementation of new ideas.
6. I am innovative.
